# Sedation and Analgesia for Toxic Epidermal Necrolysis in the Intensive Care Unit: Few Certainties, Many Questions Ahead

**DOI:** 10.3390/jpm13081194

**Published:** 2023-07-27

**Authors:** Eduardo Kattan, Maria Francisca Elgueta, Sebastian Merino, Jaime Retamal

**Affiliations:** 1 Departamento de Medicina Intensiva, Facultad de Medicina, Pontificia Universidad Católica de Chile, Santiago 8330077, Chile; e.kattan@gmail.com; 2 División de Anestesiología, Facultad de Medicina, Pontificia Universidad Católica de Chile, Santiago 8330077, Chile; panchielgueta@gmail.com; 3 Servicio de Anestesiología, Complejo Asistencial Sótero del Río, Santiago 8330077, Chile; sebastian.merino.vasquez@gmail.com; 4 Institute for Biological and Medical Engineering, Schools of Engineering, Medicine and Biological Sciences, Pontificia Universidad Católica de Chile, Santiago 8330077, Chile

**Keywords:** toxic epidermal necrolysis, intensive care unit, pain, sedation, critical care

## Abstract

Toxic epidermal necrolysis (TEN) is a rare, acute mucocutaneous life-threatening disease. Although research has focused on the pathophysiological and therapeutic aspects of the disease, there is a paucity of data in the literature regarding pain management and sedation in the intensive care unit (ICU). Most therapies have been extrapolated from other situations and/or the general ICU population. These patients present unique challenges during the progression of the disease and could end up requiring invasive mechanical ventilation due to inadequate pain management, which is potentially avoidable through a comprehensive treatment approach. In this review, we will present clinical and pathophysiological aspects of TEN, analyze pain pathways and relevant pharmacology, and propose therapeutic alternatives based on a rational and multimodal approach.

## 1. Introduction

Toxic epidermal necrolysis (TEN) is an acute mucocutaneous life-threatening disease. It is characterized by an inadequate immune response to certain triggers (mostly medications), which cause apoptosis of keratinocytes, leading to epithelial and mucosal denudation [1]. Different classifications have been proposed, being the most accepted is Bastuji-Garin, which displays Steven Johnson Syndrome (SJS), overlap syndrome (SJS/TEN), and TEN as a continuum of severity within the same disease [2]. 

Worldwide incidence has been estimated between 0.4 and 1.9 cases per million people annually, with specific high-risk populations such as HIV patients [3,4]. Mortality in different series remains between 25–30%, despite advances in diagnosis and treatment [4]. Clinically, patients present with unspecific symptoms, such as fever, headache, anorexia, and malaise, and develop a generalized rash that evolves into skin denudation, with positive Nikolsky sign, and compromising more than 30% of TBSA (total body surface area), including gastrointestinal, respiratory and genitourinary mucosas [5]. Common pharmacologic triggers include antiretroviral therapy, anticonvulsants, antibiotics, and non-steroidal anti-inflammatory drugs (NSAIDs) [5,6]. Bastuji-Garin proposed the SCORTEN, a score designed to assess the severity and probability of death of TEN patients (Table 1) [7]. It has been further used to prioritize the need for intensive care unit (ICU) admission [8,9]. 

Intense research has been directed at improving the understanding of clinical presentation, treatment, and outcome of the disease [5,10,11]. It is beyond the scope of this review to gain an in-depth analysis of these aspects.

In the ICU, adequate management of pain and sedation becomes a real challenge [12]. Considering the profound physiological derangements, multiple comorbidities and interventions, and the emotional impact on the patient, family, and ICU healthcare team, it is of paramount importance to be active and efficient in the treatment of pain. Despite this, international surveys of specialized burn ICUs have shown that, in up to 17% of TEN patients, there is no documented pain assessment [13]. This number ascends up to 50% in mechanically ventilated patients [13]. Even though mechanically ventilated patients are usually under light or deep sedation, nociception and pain in this subset of patients is a frequent and underdiagnosed issue and can lead to both physiological and psychological adverse outcomes [14,15,16,17]. Thus, adequately managing pain becomes a priority to improve clinical results [15,16].

Series show that up to 25% of TEN patients will require invasive mechanical ventilation (IMV) [8]. Among indications for IMV, authors have included unmanageable pain requiring deep sedation [9]. Mechanically ventilated TEN patients present higher organ dysfunction, morbidity, ICU length of stay, and mortality [8,9], indicating a higher severity of disease but also a higher burden of mechanical ventilation-associated complications. Improving sedation and analgesia practice could positively impact these patients by minimizing or avoiding the need for MV and its associated risks.

To the best of our knowledge, no guidelines, trials, or review articles have addressed specific analgesic and sedative therapeutic issues in TEN patients. The objective of this article is to review the pathophysiology of pain in TEN and propose an ICU sedation and analgesia algorithm based on a multimodal, physiological approach. 

For this purpose, we performed a literature search in four electronic databases (PubMED, Scopus, Web of Science, Scielo) with a combination of the following terms: “pain”, “analgesia”, “sedation”, and “toxic epidermal necrolysis”. Relevant articles written in English or Spanish were assessed for eligibility by two independent researchers (EK and SM). Differences were settled by consensus or by arbitrage by a third researcher (JR).

## 2. Insights into Toxic Epidermal Necrolysis and Pain Pathophysiology

In contrast to burn injuries, in TEN, there is no initial thermal, chemical, or electric skin insult. An immune inflammation of the dermis and mucosa (gastrointestinal, respiratory, ocular) quickly develops in a dysregulated fashion. Pathogenesis of TEN is still under study, but the latest evidence remarks the role of granulosyn, granzyme B, and perforin (three components of the cytotoxic granules of CD8+ neutrophils and Natural Killer cells, activated via MHC-I and IFN-Y pathway) as the main trigger that leads to apoptosis of keratinocytes [1,5]. 

TEN is a complex phenomenon that primarily compromises the interaction between the immunologic system and the superficial layer of the skin, which is triggered by a pharmacologic or infectious agent. It can be classified as a type IV hypersensitivity reaction, non-IgE mediated (which also includes drug reaction with eosinophilia and systemic symptoms (DRESS), Steven Johnson Syndrome (SJS), and acute generalized exanthematous pustulosis (AGEP)) [18,19,20]. Its molecular mechanism involves T-Cell receptor (TCR) interactions with human leukocyte antigen (HLA)–drug compound and leads to a reaction that has three main aspects described as apoptosis of keratinocytes via CD8 that infiltrate the skin (FAS/FASL pathway), late migration of monocytes that increase apoptotic mechanisms and necroptosis, a special apoptosis subclass [20]. Recent studies have focused on unraveling the balance between immunogenic and immunotolerant responses that could identify increased risk for these types of reactions [21].

Initially, the drug or antigen binds with transporter proteins that interact with HLA. Depending on the polymorphism the individual presents, it is more likely to develop TEN in response to a certain transporter/antigen complex, turning HLA into a possible biomarker of risk [22]. Antigen-presenting cells then interact with T-Cells through TCR. T-Cells start proapoptotic mechanisms via perforin and granzyme. Perforines are granule proteins that oligomerize into membrane-spanning pores, while granzymes are proteins that induce apoptosis per se. Granzyme function is dependent on perforin because it diffuses crossing perforin pores [23]. Additionally, granulosyn, a protein that was classically described as an antibacterial mechanism [24], also seems to have a role in the apoptotic process [20,23] due to its high intracellular titles when massive CD8 activation happens, being another possible biomarker. Moreover, this extreme inflammatory response involves massive cytokine production, and being especially important are interleukin (IL)-2, IL-15, and tumor necrosis factor alpha (TNF alpha). The latter leads to an alternative mechanism that triggers when apoptosis is blocked, necroptosis. In contraposition to apoptosis, that is, a controlled mechanism of cell degradation, necroptosis releases inflammatory mediators that contribute to maintaining inflammatory response, being a core pathological process in the TEN disease course [20].

The skin, the largest organ in human beings, has a rich innervation and pain afferents. Skin nociceptors present characteristics such as a high trigger threshold, variable intensity coding, and lack of spontaneous activity [25]. There are two main nociceptors, A-Delta and C type [25,26]. A-Delta nociceptors are comprised of fast, small-diameter myelinical nerve receptors, mainly respond to mechanical stimuli, and are located in the superficial dermis and epidermis. C-type nociceptors are slow, amyelinical nerve endings that respond to mechanical, thermal, or chemical stimuli and inflammatory mediators such as bradykinin, nitrous oxide, potassium, and acetylcholine [25].

**Figure 1:**
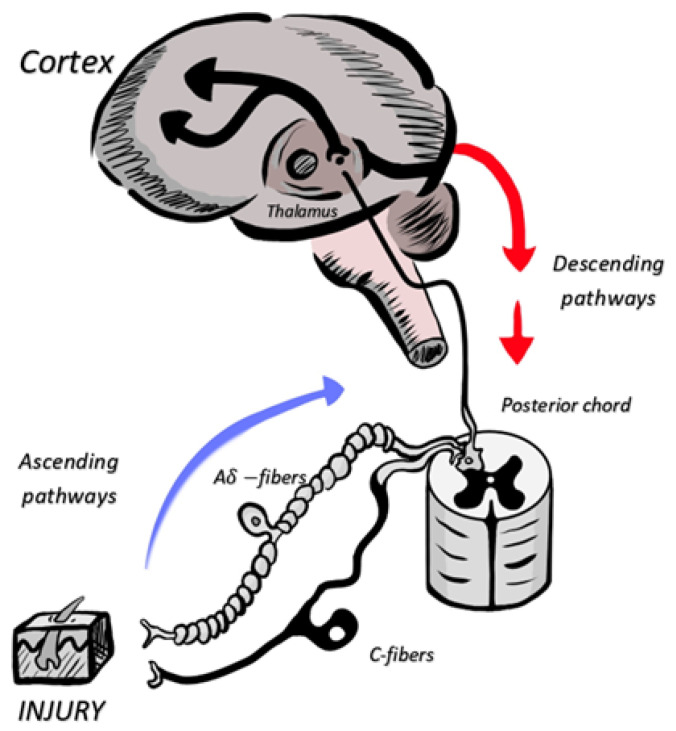
Integrated scheme of pain pathways.

Peripheral nociceptors synapse with a second neuron in the posterior ascending pathways of the spinal cord, which synapse in the thalamus where nociception is processed. From the thalamus, the third neuron projects to the cerebral cortex and amygdala, where conscious and emotional pain integration occurs [25]. Neuronal descending pathways and peripheral mediators may block, inhibit, or exacerbate the intensity of pain perception. Figure 1 illustrates relevant pathways involved in TEN-related pain. The intense activity of nociceptors and neuronal damage can reversibly modulate or irreversibly modify pain pathways [12,27]. This phenomenon leads to chronic and neuropathic pain, which can persist after the original stimulus has ceased [12,25,28]. This is why rapid, rational, and sufficient pain management is of paramount importance to minimize this process and help achieve a successful recovery [28].

A schematic represents the ascending and descending pain pathways. A-delta and C-fiber-sensitive neurons synapse with the thalamus, where a new neuronal projection transmits nociceptive stimuli to the cortex. In the cortex, these signals are integrated into the conscious experience (pain). Descending inhibitory or excitatory pathways travel through posterior cords and modulate this response.

## 3. Clinical Presentation of Pain

Pain presentation in TEN patients can be extremely variable depending on age, comorbidities, percent of total body surface area (TSBA) and specific areas compromised (including eyes and mucosa), presence of infection, and quality of pain management. Cultural and individual inputs can also alter pain perception [26,29]. 

**Figure 2:**
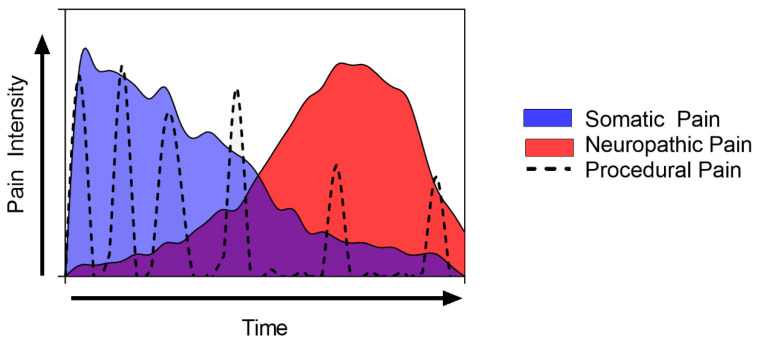
Schematic representation of toxic epidermal necrolysis’s pain evolution and characterization through time.

Besides cutaneous involvement, pain is one of the most important characteristics of TEN. Similar to other conditions, pain is presented with different patterns and temporality, as we describe in the following:

(1) Somatic pain is an intense, constant, somatic pain independent of stimulus present from the beginning of the disease, even before evident cutaneous lesions.
(2) Incidental pain can be defined as acute somatic or neuropathic pain, which has no obvious trigger and cannot be predicted [26,29]. This is one of the most difficult pain subtypes to effectively manage.
(3) Neuropathic pain can emerge as the predominant pain in the subacute phase due to neuronal damage, modulation, and modification, and a proportional decrease in somatic pain, thus becoming more apparent.
(4) Finally, procedural-related pain is an important issue to address, especially in the initial phase, in which recurrent dressing changes, debridements, and position changes are performed [30]. Figure 2 shows a scheme of the temporary evolution of pain intensity and characteristics during an ICU stay.

Adequate anxiety management is another challenge. Personal and familiar expectations, pre-procedural anxiety, and sleeping cycle alteration are some of the critical areas to be considered in the therapy. In parallel, depressive symptoms or adaptative disorders must be actively screened [31]. These patients are in constant pain, have prolonged hospitalizations, suffer deep physical derangements, and a long rehabilitation process follows hospitalization before reintegration into society. Early psychological and psychiatric evaluation is a valuable aid to help overcome this critical stage [31,32].

## 4. Multimodal Analgesic Therapeutic Approach

A multimodal and rational analgesic management strategy must try to address all pain components (somatic, incidental, neuropathic, procedural-related), include non-pharmacological and pharmacological interventions, search for synergy and minimize side effects to allow adequate recovery. 

Periodical and objective assessment is one of the most important aspects of pain evaluation. All health team members and even family can be trained to apply instruments and actively screen for pain [33,34]. The visual analog scale is the most commonly used instrument in TEN patients. Other alternatives, including face scales, anxiety scales, and sedation scales, have been validated in the ICU setting. For pain assessment in ventilated patients, the Critical Care Pain Observation Tool (CPOT) and Behavioral Pain Scale (BPS) are the most common and validated alternatives [14]. Novel monitors for nociception assessment in ventilated ICU patients, such as ANI^®^ (MDoloris Medical Systems, Loos, France) and NOL^®^ (Medasense Biometrics Ltd., Ramat Gan, Israel) [16,35], integrate objective physiological signals and have been used for research purposes [16]. These could soon be transferred to clinical practice in the future and help titrate analgesic therapy.

Non-pharmacological interventions can be varied and tailored to specific patient needs. Despite a lack of solid evidence, these interventions are low-cost, easily implemented, and with a positive impact on the quality of care. Some interventions used in TEN [13] and other contexts include prolonged family visiting hours [36], music therapy [37], adequate room temperature [6], and a multidisciplinary approach, including physical therapy, speech therapy, psychologists, and occupational therapy [32]. Topical lubricants and coverage in skin, mucosa, and eye (including contact lenses) prevent itching, dryness, and pain and are of valuable aid [6,38,39,40]. 

Immunosuppressive drugs (corticosteroids, intravenous immunoglobin, ciclosporin, and biological agents) [5,10,41] have been fostered as the main therapeutic alternatives to treat TEN [42]. The main objective is to prevent disease progression by modulating the inflammatory response. Interestingly, it could also have a role in decreasing painful stimuli, acting at different cellular levels: modulating CD8 migration and activation [10,43], inhibiting keratinocyte apoptosis [43], and decreasing inflammatory response directly on cells exposed to intracellular content released from skin cells destroyed by granulosyn [44,45]. The specific impact of immunomodulators on pain should be further addressed by clinical and physiological trials.

Multimodal pharmacologic analgesic interventions provide a synergistic effect, diminishing dosage, cumulative adverse effects, dependence, and potentially reducing pain chronification. Table 2 presents a safe starting dose for each pharmacological intervention proposed. Intravenous opioid therapy remains one of the main pillars of treatment. Morphine, Fentanyl, and Methadone (*u*-receptor agonists) provide effective analgesia for somatic and visceral pain with no ceiling effect. Methadone, additionally being an NMDA receptor agonist, has the theoretical advantage of acting in dual pain pathways (somatic and neuropathic) and diminishing the long-term adverse effects of opioid therapy [46]. 

Patient-controlled intravenous analgesia (PCEA), when feasible (taking into consideration the ability to effectively push the button), allows the patient to have higher control of pain management and a better VAS score when compared with an on-demand or hourly schedule [47]. Adverse effects of opioids include respiratory depression, dependence tolerance, hyperalgesia, pruritus, and constipation. If available, prompt evaluation by a pain specialist can aid in developing a treatment plan.

Co-adjuvant therapy includes low-potency analgesics, such as acetaminophen, which are helpful in somatic and visceral pain control and have opioid sparring effects. Ketamine, an NMDA receptor antagonist, is a safe and potent analgesic with neuropathic and somatic analgesic effects, and can be used in continuous infusion and in on-demand bolus rescues [48]. ICU and intraoperative studies show that ketamine infusion diminishes up to 30% of opioid requirements [49]. Ketamine has sedative properties, usually described as a dissociative unconsciousness. Side effects include sialorrhea, tachycardia, vivid dreams, and hallucinations [48,49]. 

As mentioned before, neuropathic pain becomes one of the therapeutic challenges in TEN patients. For neuropathic pain, we usually perform a lidocaine test, which consists of the administration of 1 mg/kg of lidocaine in a 60 s bolus. The test is considered positive if pain diminishes by 50% or more [54]. With a positive test, lidocaine infusion and pregabalin are started [54]. Chlorphenamine is a histamine H1-receptor antagonist with sedative and anti-pruriginous properties, useful for treating pruritus due to denudation and re-epithelization.

Sedation must focus on anxiety relief but also allow patients to maintain airway reflexes and breathe easily without airway obstruction. There is a vast therapeutic arsenal that can be used in the ICU setting. Dexmedetomidine, an alpha 2 agonist, is an interesting alternative because it has sedative, analgesic, and anxiolytic effects, is easily titratable, has a modulatory adrenergic effect, has no respiratory depression, and has a short elimination half-life [51]. Clonidine, a less potent alpha 2 agonist, can be used orally to pursue similar results [57]. Benzodiazepines, as well as Midazolam or Lorazepam, are a valuable treatment option when dexmedetomidine is not available or is contraindicated [53]. Concerns about the higher incidence of delirium, extrapolated from mechanical ventilation studies, must be balanced versus potential benefits [52]. 

Selective serotonin reuptake inhibitors or serotonin–noradrenalin reuptake inhibitor (dual) antidepressants can help manage depressive symptoms in the ICU [55]. Dual antidepressants have the added benefit of proven efficacy against neuropathic pain [56]. Finally, for intra-ICU procedures, which need a higher level of sedation, propofol infusion, optimally delivered with a Target Controlled Infusion associated with potent opioids, can be initiated [50]. Concerns about hypotension, airway patency, and hypoxemia must be balanced with adequate sedation and analgesia.

**Figure 3:**
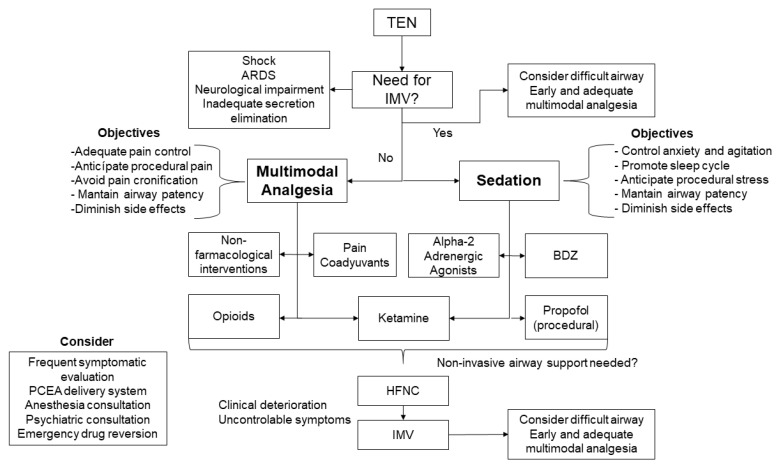
Proposed multimodal therapeutic algorithm.

TEN patients in the ICU present a real challenge, considering the pharmacokinetic and pharmacodynamical derangements, changes in the volume of distribution, polypharmacy, and organ failures, among others. In this line, clinicians should be cautious of potential drug interactions (such as serotoninergic syndrome), and cross-pharmacological reactions (including suspected triggers for TEN) must be considered because continuous exposure to triggers could lead to worse clinical results [58]. Thus, consultation with clinical pharmacists could become of valuable aid in avoiding known and unwarranted adverse effects. Figure 3 depicts the proposed algorithm to address sedation and analgesia in the setting of TEN patients.

## 5. Potential Adverse Reactions

Tailored sedation and analgesic therapy can be difficult to achieve, even more so when potential adverse effects could determine serious negative outcomes. As with any ICU patient, basic and advanced monitoring, along with frequent visual inspection, must be implemented (even though sometimes it provides a real challenge). Teams must be prepared to rapidly diagnose and treat over-sedation and opioid adverse reactions such as respiratory depression, hypoventilation, superior airway obstruction, and hypoxemia. Treatment should include immediate suspension of sedatives, drug reversion with naloxone and flumazenil, oxygen supplementation, or even invasive ventilation. 

Traditional non-invasive ventilation (NIV) has been regarded as a relative contraindication for burn patients with facial wounds [59], and the case series shows that it is seldom used in TEN patients [9]. High flow nasal cannula (HFNC) appears as an interesting alternative to prevent airway complications in this setting, considering it has a smaller contact surface area than NIV, and is better tolerated by patients. Positive physiological effects include the moistening and warming of air/oxygen, effective low PEEP level, and wash-out of CO_2_ [60]. If invasive airway management is needed, TEN patients must be considered to have difficult airways, and all due measures must be taken into consideration [30,61].

Ketamine hallucinations and vivid dreams can be mitigated with dexmedetomidine or benzodiazepines, with good results [48]. Opioid tolerance refers to a lack of clinical response with increasing dosage, while opioid-induced hyperalgesia refers to a paradoxical increase of pain in response to opioid administration. Opioid dependence refers to the clinical and psychological symptoms developed after the suspension of chronic therapy. Management of these aspects is beyond the scope of this review, but multiple reviews address the physiopathology, diagnosis, and treatment [62]. Moreover, because patients with TEN are usually exposed to opioids for prolonged periods of time and in moderate to high dosing schemes, multimodal analgesic strategies as those previously mentioned and prompt de-escalation could potentially have the added benefit to reducing overall exposure to narcotics, and thus, decrease the incidence of opioid dependence in this fragile group of patients. Complementing these therapies with a multidisciplinary rehabilitation (i.e., physiotherapists, speech therapists, mental health professionals, among others) approach could further reduce this side effect.

## 6. Global Aspects and Future Directions

Management of TEN patients in the ICU is a complex challenge, which involves significant morbidity and mortality, prolonged hospitalizations, and high demands of healthcare expenditure, which can potentially burn out the ICU team. Additionally, personal and familiar expectations must be addressed and aligned with potential outcomes. As for pain management, the ICU team must be trained in periodical evaluation and consider it a fundamental aspect of the patient’s therapy, in which everybody involved can intervene. As we mentioned before, a multi-professional team should be actively engaged in providing adequate sanitary care. Even though much of the therapy implemented is extrapolated from burn and general ICU patients, future studies should be performed in the specific context of TEN patients.

Despite it being time-consuming and can be impracticable in some scenarios, tailored sedation strategies by experienced providers (anesthesiologists, ICU nurses, or physicians), adequate monitoring and planning can avoid the need for invasive mechanical ventilation and enable the performance of painful procedures while spontaneously breathing. As mentioned before, the need and duration of invasive mechanical ventilation have been associated with increased ICU length of stay, systemic and respiratory complications, and even death [63]. 

## 7. Conclusions

TEN is a rare but life-threatening disease. Patients present with high and persistent pain, and due to their complexity, pain treatment, unfortunately, is sometimes considered a second-class priority. We present an analysis of the physiopathology of pain in TEN patients and propose a rational and multimodal therapeutic approach. We believe that, after all, the most important aspect to successfully managing TEN patients is a committed ICU interdisciplinary team who can implement non-pharmacological and pharmacological measures tailored to the patient’s needs and, most importantly, can provide humane and empathic care to the suffering patient and family in front of them.

## Figures and Tables

**Table 1:** SCORTEN variables and predicted hospital mortality.

SCORTEN	Mortality Rate % (95% Confidence Interval)
0–1	3.2 (0.1–16.7)
2	12.1 (5.4–22.5)
3	35 (19.8–53.5)
4	58.3 (36.6–77.9)
≥5	90 (55.5–99.8)

Variables included: age > 40 years, presence of malignancy, heart rate > 120 beats per minute at admission, body surface area detached >10%, serum urea > 10 mmol/liter, serum glucose >14 mmol/liter, and serum bicarbonate < 20 mmol/liter. Adapted from reference [7].

**Table 2:** Proposed multimodal therapeutic approach for patients with TEN in the ICU.

Pain Component	Drug	Safe Starting Dose	Maximum Dose	Special Considerations
Background pain	Methadone bolus [46]	0.05 mg/kg	Dosage titration until side effects appear (i.e., respiratory depression, sedation)	Renal excretion Decrease dose to 50% with glomerular filtration rate < 10 mL/min QT segment prolongation
	Morphine bolus [14,30]	0.05 mg/kg	Dosage titration until side effects appear (i.e., respiratory depression, sedation)	Renal excretion Avoid in case of acute or chronic kidney injury
	Fentanyl bolus [14,30]	25–50 mcg	Dosage titration until side effects appear (i.e., respiratory depression, sedation)	Respiratory depression
Breakthrough pain	Rescue opioid PCEA [47]	Use according to local algorithm	Dosage titration until side effects appear (i.e., respiratory depression, sedation)	Consciousness level for adequate use, hands must be available for control
	Ketamine bolus [48,49]	0.25 mg/kg	0.35–0.5 mg/kg	Hallucinations, salivation Use with caution in intracranial hypertension
Pain co-adjuvants	Ketamine infusion [49]	0.25–0.5 mg/kg/h	1–2 mg/kg/h	Hallucinations, salivation Use with caution in intracranial hypertension
	Paracetamol [14,39]	1 g c/8h	1 g c/6 h	Risk of hepatotoxicity Avoid in case of fulminant hepatic failure
Procedural sedation	Propofol [14,50]	0.2–4.8 mg.kg^−1^.h^−1^	Titrate dosage until the clinical sedation objective achieved	Oversedation, propofol infusion syndrome (PRISS), hypertriglyceridemia
Anxiety	Dexmedetomidine [51,52]	Start at 0.2 mcg kg^−1^ h^−1^ and adjust according to the response	1.4 mcg kg^−1^ h^−1^.	Contraindicated in refractory hypotension and second or third-degree heart block
	Lorazepam bolus [53]	2 mg bolus	N/A	Delirium, accumulation
	Midazolam infusion [53]	2–5 mg/h	N/A	Delirium, accumulation
Neuropathic pain	Lidocaine infusion [54]	4 mg/kg to infuse in 4 h	-	Cardiac conduction alterations, local anesthetic systemic toxicity
Depressive symptoms	Duloxetine [55,56]	30 mg c/24 h	120 mg/day	Do not use concomitant to IMAO Avoid in glaucoma
	Venlafaxine [55,56]	37.5 c/24 h	150–225 mg/day	Renal adjustment requiredDo not use concomitant to IMAO
Anticonvulsants	Pregabalin [56]	25–150 mg c/24 h	150–300 mg BID	Renal adjust required
	Gabapentin [56]	100–300 mg/24 h	300–1200 mg TID	Renal adjust required

TEN: toxic epidermal necrolysis. ICU: intensive care unit. PCEA: patient-controlled endovenous analgesia. N/A: not applicable. IMAO: inhibitor of monoamine oxidase.

## Data Availability

All data generated or analyzed during this study are included in this published article.
